# Leptomeningeal Carcinomatosis in a Young Patient With Colorectal Cancer: A Case Report

**DOI:** 10.7759/cureus.68519

**Published:** 2024-09-03

**Authors:** Zamanali Khakhar, Soraiya Manji, Rajiv Patel, Karishma Sharma, Sheila Waa, Sayed K Ali

**Affiliations:** 1 Department of Medicine, University of Nairobi, Nairobi, KEN; 2 Department of Internal Medicine, Aga Khan University Hospital, Nairobi, KEN; 3 Department of Internal Medicine, Avenue Healthcare, Nairobi, KEN; 4 Department of Radiology, Aga Khan University Hospital, Nairobi, KEN

**Keywords:** neoplastic meningitis, colon cancer, carcinomatous meningitis, colorectal cancer (crc), leptomeningeal carcinomatosis (lmc)

## Abstract

Leptomeningeal carcinomatosis (LMC) is a rare and fatal complication associated with various solid tumors and hematological malignancies. It presents significant diagnostic challenges due to its nonspecific symptoms and complex clinical course. This case report details the presentation, diagnosis, and implications of LMC in a 33-year-old male with a history of colorectal cancer (CRC) and hemicolectomy. The patient presented with nonspecific symptoms of headache and dizziness. Cerebrospinal fluid cytology revealed the presence of malignant cells, leading to the diagnosis of LMC secondary to CRC. The elusive and multifaceted nature of this condition highlights the necessity for increased clinical awareness and extensive research to improve the understanding and management of LMC.

## Introduction

Leptomeningeal carcinomatosis (LMC), also referred to as carcinomatous meningitis, is a rare and severe condition characterized by disseminated malignant cells within the pia and arachnoid mater, collectively known as the leptomeninges. It is a devastating complication associated with several solid tumors (breast cancers, brain cancer, and melanomas), lymphomas, and hematological cancers that have the potential to metastasize and involve the meninges. LMC usually occurs as a terminal, late-stage complication of these cancers and is often associated with a poor prognosis. LMC in the setting of colorectal cancer (CRC) is extremely rare and has an incidence of less than 1% [1]. Early diagnosis of LMC, particularly before the onset of neurological deficits, enables timely intervention, which may lead to more effective treatment and an improved quality of life for patients [2]. CRC ranks as the third most prevalent cancer among males and second among females. It is the fourth leading cause of cancer-related death in males, while in females, it ranks third [3]. Metastasis to the leptomeninges can occur through various routes, resulting in LMC. The deficiency of information on LMC in the context of CRC and the low prevalence highlights the importance of increased awareness and the necessity for advanced diagnostic approaches to effectively identify and manage LMC.

In this case report, we seek to elucidate this rare complication in a 33-year-old male with a history of CRC, previously managed with hemicolectomy. We explore the complexity of its presentations, clinical courses, diagnostic challenges, and therapeutic modalities that may be implemented to improve the survival of the patient.

## Case presentation

A 33-year-old male of African descent with a prior history of colon cancer for which he had a right-sided hemicolectomy in November 2023, presented to our facility a few months later for a persistent and severe headache associated with dizziness. He was accompanied by his sister who reported that the headaches had commenced a few weeks prior to his presentation. The headaches were non-radiating in nature, exacerbated by a change in position particularly when sitting up, and alleviated by lying down. Additionally, the headaches were associated with intermittent episodes of nausea and vomiting. No fevers were reported; however, there were instances of intermittent confusion that had been progressively worsening. He had no other pre-existing medical conditions and did not engage in the use of tobacco, alcohol, or illicit or recreational substances. The patient tested negative for human immunodeficiency virus (HIV).

The physical exam revealed a cachectic young man holding his head within his palms in obvious pain. He was unable to follow commands but demonstrated purposeful movement in response to painful stimuli. His heart and lung exams were within normal limits. His abdominal exam showed a laparotomy scar but was soft to touch with positive bowel sounds. The patient had a Glasgow Coma Scale (GCS) score of 14/15. His cranial nerve, motor, sensory, and cerebellar functions were all intact.

In the assessment of the patient's condition, laboratory results were acquired and are presented in Table 1.

**Table 1:** Patient laboratory results. GFR: glomerular filtration rate; CEA: carcinoembryonic antigen.

Tests	Results	Normal range
White blood cell count	9.18 × 10^9^/L	4.5 - 11.0 × 10^9^/L
Hemoglobin	14 g/dl	14 - 18 g/dl
Hematocrit	43%	41 - 50%
Platelets	153 × 10^9^/L	150 - 400 × 10^9^/L
Sodium	139 mmol/L	135 - 145 mmol/L
Potassium	4.69 mmol/L	3.6 - 5.2 mmol/L
Creatinine	81 µmol/L	72 - 127 µmol/L
GFR	113 mL/min/1.73 m²	80 - 120 mL/min/1.73 m²
CEA	25.93 ng/ml	<2.5 ng/ml (non-smoker) or <5.0 ng/ml (smoker)

**Figure 1:**
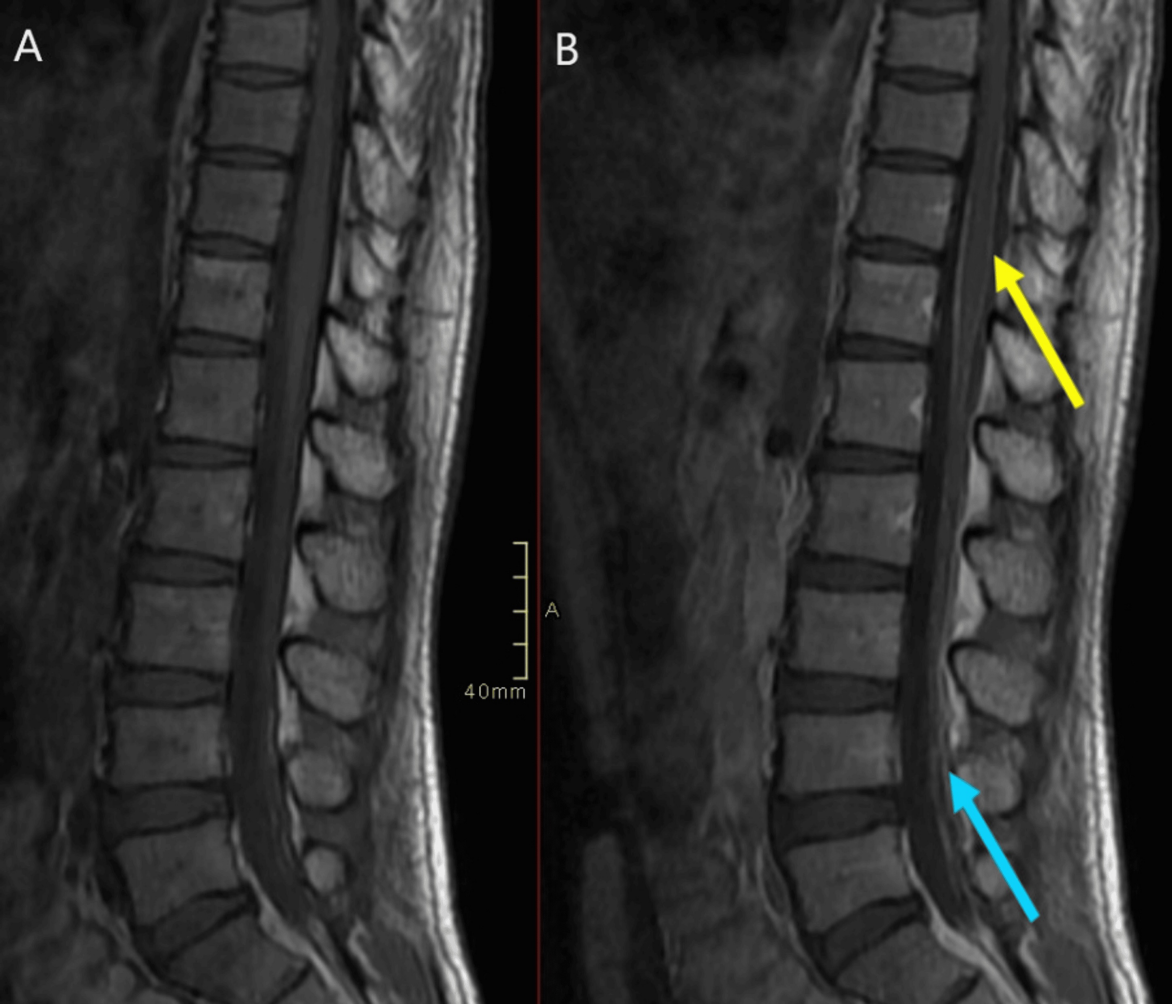
Pre- (A) and post-contrast (B) sagittal T1 images demonstrating enhancement of the conus medullaris leptomeninges (yellow arrow) and of the cauda equina (blue arrow).

**Figure 2:**
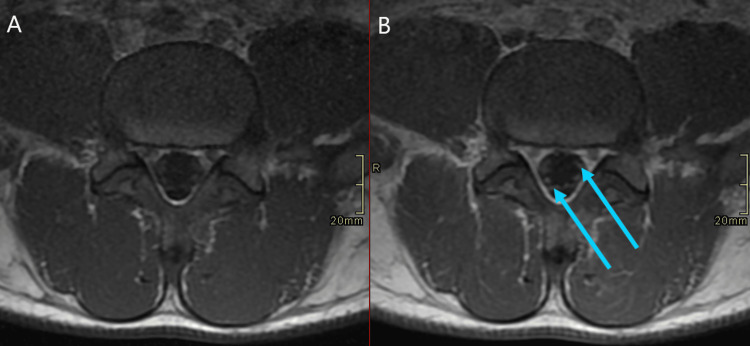
Pre- (A) and post-contrast (B) axial T1 images of the lumbar spine demonstrating uniform enhancement of the cauda equina ventral and dorsal nerve roots (blue arrows).

A positron emission tomography (PET) scan was performed, revealing that the patient had undergone a right hemicolectomy, with low-level fluorodeoxyglucose (FDG) uptake in the regional right mesenteric lymph nodes and aortocaval lymph nodes. When compared with the previous PET-CT done four months prior, there was an interval increase in size and number of the low level of nodes suggesting disease progression. MRI of the brain showed no acute intracranial findings, but an MRI of his spine (Figure 1) showed enhancement of the conus medullaris leptomeninges and cauda equina nerve roots, both ventral and dorsal (Figure 2), suggestive of either meningitis or carcinomatosis given the patient's underlying condition.

The patient's lumbar puncture revealed several abnormalities, most notably an elevated opening pressure and an increased cerebrospinal fluid (CSF) white blood cell (WBC) count. The CSF analysis indicated an opening pressure of 40 cmH_2_O, which is significantly higher than the normal range of 10-20 cmH_2_O. The findings are summarized in Table 2.

**Table 2:** Cerebrospinal fluid (CSF) analysis results.

Tests	Results	Normal range
Color	Clear	Clear
Opening pressure	40 cmH_2_O	10 - 20 cmH_2_O
CSF white blood cell count	78/mm³	0 - 5/mm³
Lymphocytes	98%	62% ± 34
Neutrophils	2%	2% ± 5
CSF glucose	2.60 mmol/L	2.8 - 4.2 mmol/L
CSF protein	0.19 g/L	0.15 - 0.45 g/L

His CSF viral panel was negative for cytomegalovirus (CMV), influenza virus, *Neisseria meningitidis*, and human herpesvirus 6 (HHV-6). His tuberculosis (TB) polymerase chain reaction was also reported as negative. Notably, his CSF cytology revealed the presence of malignant cells, thereby confirming the diagnosis of leptomeningeal carcinomatosis secondary to colon cancer.

Our oncology team offered the patient intrathecal chemotherapy via an Ommaya reservoir as well as systemic chemotherapy, but the patient and his family declined treatment opting to return back home to Uganda. Despite multiple attempts to contact the patient to assess the status of the disease, there was no response and his phone had been disconnected, therefore post-discharge information about the patient is unavailable.

## Discussion

LMC occurs as a rare and catastrophic complication of CRC. Despite the advancement in cancer therapeutics, mortality remains high. The scarcity of documented cases of LMC secondary to colorectal carcinoma in the literature highlights the significant deficit in knowledge regarding the possibility of such a complication and the diagnostic and therapeutic challenges that accompany this condition. Therefore, this case report emphasizes the need for increased vigilance and diligence in managing patients who exhibit neurological symptoms, particularly when LMC is suspected. It further aims to contribute to the currently scarce medical literature on LMC secondary to CRC by offering new insights into the presentation and diagnosis thereby enhancing disease understanding and aiding healthcare professionals in similar cases. Documenting such a case can stimulate further research into this condition.

The incidence of clinically diagnosed LMC among patients with solid tumors is estimated to be approximately 5%. While any cancer can metastasize to the meninges, the most common sources of solid tumor-related LMC are breast cancer (12-35%), lung cancer (10-26%), melanoma (5-25%), gastrointestinal cancer (4-14%), and cancers of unknown primary origin (1-7%) [2]. LMC is extremely infrequent in colorectal carcinoma, with studies suggesting an occurrence rate of less than 0.02% [3]. This accentuates the poorly understood aspect of cancer metastasis in this context. Even as cancer therapeutics advance, the prognosis for patients with LMC remains very poor, with a median overall survival of two to four months with the currently available treatments and one to 1.5 months without treatment [4].

Depending on the origin of the tumor, malignant cells can spread through various routes, including hematogenous spread through venous plexi, direct seeding from brain parenchyma (by breaching the meninges and entering the CSF), or traversing the fenestrated endothelium of the choroid plexus to eventually seed the meninges [5]. The malignant cells enter the CSF and disseminate over the surface of the meninges. The malignant cells disperse through the CSF to distant sites of the central nervous system (CNS) where they implant and grow to form secondary leptomeningeal metastatic deposits, seeding into the leptomeninges focally or diffusely. Diffuse covering of the leptomeninges is frequently observed in hematologic malignancies, whereas plaque-like deposits and nodular formations are more commonly associated with solid tumors. The brain, spinal cord, and spinal roots may appear normal upon gross examination. More frequently, the leptomeninges exhibit changes such as thickening and fibrosis that may be localized or widespread in certain sites within the CNS especially where CSF stasis is prominent [2].

A myriad of signs and symptoms characterize LMC, which include headache, diplopia, confusion, cognitive impairment, nausea, and vomiting. Involvement of cranial nerves and spinal cord can result in further symptoms due to compression. Common cranial nerves involved include abducens and facial nerves, compression of which results in diplopia and facial paresis, respectively. Spinal manifestations associated with LMC are back or neck pain, limb paraesthesia, lower motor weakness, radiculopathy, and bladder and bowel dysfunction. Elevated intracranial pressure caused by impaired CSF reabsorption can manifest as headache, nausea, and vomiting [5].

Diagnosing LMC poses a significant challenge due to its nonspecific symptoms and the rarity of this condition as a complication of CRC. Due to the ambiguous presentation, it is essential to rule out other alternative diagnoses that may include primary brain tumors, stroke, meningitis, encephalitis, brain abscesses, etc. The currently available diagnostic modalities have low sensitivity. Gadolinium-enhanced MRI is the preferred imaging technique for LMC due to its high sensitivity (59%) and specificity (95%) as compared to CT scans [3]. CSF cytology can be used as a diagnostic method, with 75% sensitivity and 100% specificity [6]. Subsequent lumbar punctures increase the sensitivity to 100% after three attempts, thus initial negative findings do not rule out the diagnosis of LMC [7]. Immunohistochemistry reveals molecular markers associated with LMC, which include E-cadherin-catenin complex, plasmin, urokinase-type plasminogen activator, and metalloproteinase [6,8].

In the current case, CSF cytology was positive for malignant cells and aided in the diagnosis of LMC as the clinical signs and symptoms were not conclusive for LMC. With the majority of case reports in the literature on LMC focusing on more common primary malignancies such as breast cancer, lung cancer, and melanoma, our case report diverges from these as it addresses LMC as a complication of CRC, a rare occurrence.

The patient was offered chemotherapy via an Ommaya reservoir to slow the progression of the disease with an attempt to potentially prolong survival as much as possible while also preventing the progression of neurological symptoms. The Ommaya reservoir, invented by Dr. Ayub K. Ommaya, is a ventricular access device equipped with an intraventricular catheter designed for prolonged intrathecal access [9]. It facilitates the delivery of medications, such as antineoplastic drugs to the CSF efficiently and can remain in place for months hence it was the apparatus of choice recommended by the oncologist in this case.

Due to the high mortality rate associated with LMC, the principal therapeutic goal is to provide optimal palliative care with extending survival as a secondary objective. However, novel therapies currently under trial are aimed at improving survival and delaying neurological progression, offering a more hopeful outlook for patients afflicted with this condition. Intrathecal formulations of trastuzumab, nivolumab, and pemetrexed have shown favorable safety profiles and have been associated with improved median survival rates of four to nine months in recent phase I and II clinical trials [10]. Radiation therapy is a key treatment modality for leptomeningeal metastasis. The approaches employed include whole-brain radiotherapy (WBRT) and involved-field radiotherapy, primarily used for palliative care. Additionally, craniospinal irradiation (CSI) and proton craniospinal irradiation (pCSI) are used, with the latter providing more precise targeting and fewer side effects [11]. The primary consideration is the improvement of quality of life with analgesics, antidepressants, and other psychotherapeutics. Reduction of intracranial pressure by relieving obstruction in the CSF flow surgically may be of some benefit. Novel therapies can also be administered as exploratory options with the purpose of prolonging survival. The prognosis remains poor. Even with treatment, mortality occurs within two to four months from the onset of symptoms. A multidisciplinary approach is needed to achieve optimal care of the patient in a holistic fashion.

Further research is warranted to explore the pathophysiology of LMC, to determine if certain individuals are more susceptible to developing LMC as a complication of CRC than others, and to investigate whether a genetic or racial predisposition may influence the emergence of this complication. Additionally, novel pharmacological interventions for LMC should also be aimed at improving the quality of life, prolonging survival, and slowing the progression of the disease. Attending physicians should be aware and vigilant of this rare complication of CRC so as to initiate early response and management.

## Conclusions

LMC represents a severe and fatal complication of CRC. In patients presenting with neurological symptoms and a primary cancer diagnosis, it is crucial to consider LMC as a differential diagnosis. The diagnostic difficulties and the currently limited treatment options confer significant challenges in managing LMC effectively. Given the poor prognosis, rapid progression, and imminent mortality frequently documented in the literature, palliative treatment remains the standard of care for patients with LMC. Future research must prioritize the development of comprehensive diagnostic protocols and therapeutic algorithms that facilitate the swift diagnosis and early management of patients with LMC. Exploring novel treatment modalities and improving symptom management strategies can significantly improve patient outcomes. Advancing research in these areas can enable clinicians to better navigate the complexities and intricacies of LMC.
